# Pivot Crown

**Published:** 1884-07

**Authors:** F. E. Howard

**Affiliations:** Buffalo, N. Y.


### "PIVOT CROWN."

BY F. E. HOWARD, M. D. S., BUFFALO, N. Y.

I have devised a new method of setting the "Bonwill Pivot Crown," and others of its class, which to my mind is far superior to the system advised by Dr. Bonwill, viz, the use of amalgam in making the setting, which I have long since abandoned.

After the usual preparation of the root, select platinum or good wire, barbed, of proper size, or a Bonwill pin. Make a mixture of zinc phosphate, fill the root, crowd the pin in position, and impact the cement to pin and root. The pin should be long enough to extend into the opening of the pivot crown on the palatine surface.

After the cement sets, trim down all excess of filling to a flat surface. Take of good gutta percha a piece that will cover the end of the root, and the thickness of card-board; punch a pin-hole in it and adjust it to the root. Make the second mixture of cement and fill the crown. Slightly warm the gutta percha, put it in position, and crowd the crown to its place.

By this method a joint is secured that is impervious to moisture, preventing disintegration of the cement, which is very strong and all that can be desired if kept from the secretions of the mouth.

Now, if you desire it to be still more permanent in its character, cut out the excess of cement at the palatine cavity, and fill with gold or amalgam, bringing the filling in contact with and partly anchoring it to the pin that extends into the root. This method gives us a pivot crown beautiful in appearance, and practically as strong as any that we have.

Should any accident necessitate repair, a new crown is easily adjusted.

